# A Cox Proportional Hazards Model Approach to Identifying Malaria Risk Factors in Children in South Sudan

**DOI:** 10.7759/cureus.99083

**Published:** 2025-12-12

**Authors:** Loro Gore Lado Jumi, Altaiyb Omer Ahmed Mohmmed

**Affiliations:** 1 Department of Statistics and Demography, School of Social and Economic Studies, University of Juba, Juba, SSD; 2 Department of Statistics, College of Science, Sudan University of Science and Technology, Khartoum, SDN

**Keywords:** cox proportional hazards model, hazard ratio, malaria, partial likelihood, schoenfeld residuals

## Abstract

Introduction

Malaria represents a critical public health concern worldwide, with a pronounced impact in tropical and subtropical regions. In South Sudan, it is a primary contributor to illness and death, particularly among children. This study aimed to identify the risk factors that affect the survival of children with malaria in South Sudan.

Methods

This hospital-based retrospective study was carried out at Al Sabah Children’s Hospital in Juba, South Sudan. Data were obtained from the records of children with malaria who were admitted and ranged in age from 1 month to 15 years during the period from January 1 to December 31, 2021. The Cox proportional hazards model was employed to identify the risk factors affecting the survival of children suffering from malaria.

Results

This study included 6,410 children diagnosed with malaria; males constituted 56.08% and females 43.92%. Censored cases were 95.27%, and those who died constituted 4.73%. The average age of the study cohort was 23.66 months (95% CI: 23.01 to 24.32). Results from the Cox proportional hazards model showed that age was associated with an increased hazard of death (hazard ratio 1.0223, 95% CI: 1.0158 to 1.0280), and body weight was associated with a decreased hazard of death (hazard ratio 0.8513, 95% CI: 0.8146 to 0.8896). Treatment with quinine was associated with a lower risk of death (hazard ratio 0.7107, 95% CI: 0.5501 to 0.9183) compared to treatment with artesunate. Children without comorbidity had a lower risk of death (hazard ratio 0.5530, 95% CI: 0.4273 to 0.7157) compared to those with comorbidity. These covariates were statistically significant (p < 0.01).

Conclusion

In this study, increased mortality risk was linked to both age and the presence of comorbidities among children with malaria. In contrast, increased body weight reduces the hazard of death. Also, treatment with quinine is associated with a reduced risk of death compared to artesunate, which is contrary to studies showing that artesunate is superior to quinine. Quinine is still the drug of choice in the treatment of malaria in South Sudan. Enhancing programs aimed at reducing the malaria burden in children, and ensuring the availability of good-quality drugs to improve treatment outcomes, is important for child health.

## Introduction

Despite huge investments and efforts to respond to malaria, the disease continues to pose a significant public health issue globally, especially in tropical and subtropical areas. In 2019, around 227 million cases of malaria were reported across 85 countries where the disease is prevalent. In 2020, following the COVID-19 pandemic and the disruption of healthcare services, the estimated number of malaria cases increased to 241 million. Additionally, between 2019 and 2020, the estimated number of malaria deaths increased by 12%, from 558,000 to 627,000. In the WHO African Region, a significant portion of malaria cases and deaths occur, with 80% of these deaths affecting children under five years of age [1].

Malaria is a serious and fatal disease caused by *Plasmodium* parasites, which are transmitted to humans via bites from infected female *Anopheles* mosquitoes [2]. This disease mainly affects children who lack sufficient immunity. Ineffective or delayed treatment may result in the progression to severe malaria and potential death. In addition to the immediate risk to life, malaria in children can cause long-lasting effects, including hindering cognitive development and compromising overall health and well-being. Moreover, infections in children serve as a reservoir for further parasite transmission, which hampers progress in malaria prevention and control [3]. The high prevalence of malaria in Sub-Saharan Africa is attributed to a combination of broader issues, such as insufficient financial resources, poverty, resistance from parasites and mosquitoes, weak healthcare systems, and general attitudes toward seeking medical care [4]. In a study on public health determinants of child mortality in Kenya, the Cox proportional hazards model was used to model time to death. The results revealed that the hazard of death was higher among the youngest children compared to older children [5].

South Sudan is among the countries with the highest malaria transmission. Transmission occurs throughout the year, with peaks between July and November. *Plasmodium falciparum* is the main species, accounting for most infections. Malaria is endemic in all regions of the country and is a significant contributor to illness and death, particularly in children. In 2021, South Sudan was identified as one of the 22 countries experiencing the highest malaria burden worldwide, representing 1.2% of global malaria cases and fatalities. In East and Southern Africa, South Sudan contributed 5% of malaria cases [1]. The study by Jumi [6] on the trend of malaria incidence in Jubek State, South Sudan, using generalized linear models, showed that malaria incidence was increasing. The incidence rate suggested an increase of 0.3% per week. According to Huang et al. [7], the disease is responsible for approximately 66.8% of visits to outpatient departments in health facilities, 30% of all hospital admissions, and 50% of fatalities occurring in hospitals. Studies on malaria in South Sudan have focused on malaria prevalence, control intervention measures, and the clinical management of cases. Studies examining risk factors associated with malaria are lacking. Therefore, this study aimed to determine malaria risk factors that affect the survival of children with malaria in South Sudan.

## Materials and methods

This retrospective study was carried out at Al Sabah Children's Hospital in Juba, South Sudan. The data were collected from the records of children ranging in age from 1 month to 15 years who were admitted between January 1 and December 31, 2021. Children with missing data on the variables were excluded. In this study, the response variable was the duration a child with malaria survived. The covariates considered were age, gender, body weight, season, location of residence, treatment, and the presence of another disease. To identify the risk factors influencing the survival of children with malaria, the Cox proportional hazards model was employed.

Description of variables 

The continuous variables encompass the survival time, defined as the period from a child's hospital admission to death due to malaria or censoring, expressed in days. Additionally, the age of a child diagnosed with malaria is recorded in months, and body weight is measured in kilograms. The categorical variables include the child's gender, coded as 1 for male and 0 for female. Furthermore, the season is categorized, with the dry season occurring from December to March and the wet season from April to November, coded as 1 for wet and 0 for dry. Treatment was coded 1 for quinine and 0 for artesunate. Place of residence was coded 1 if urban and 0 if suburban. Comorbidity status was coded 1 if one or more additional diseases were present and 0 if not. All data analyses were performed using STATA version 14.1 Special Edition (StataCorp LLC, College Station, TX, USA). 

Ethical considerations

Approval for the study was granted by the Ministry of Health's Research Ethics Review Board (MOH/RERB) in Juba, Republic of South Sudan, under approval number MOH/RERB 55/2022, dated September 30, 2022. The data gathered were anonymized, and patients’ confidentiality was maintained.

Survival analysis

Survival analysis involves a variety of statistical methods designed to investigate data concerning the time until a particular event occurs. This event could be death, the onset of a disease, the recurrence of a disease, or any other specified event of interest that might happen to an individual [8]. A key aspect of survival analysis is censoring, which occurs when the exact time of an event of interest for an individual is unknown. Censoring may occur for several reasons, such as when the event of interest has not happened to an individual by the end of the study, when a patient is lost during follow-up, or when they withdraw from the study. The analysis of survival relies on the distribution of survival times. The distribution is characterized by two fundamental functions: the survival function and the hazard function.

Survival function

The survival time, represented by $t$, is an observed value of a random variable $T$ which can only assume nonnegative values. Assume that $T$ follows a probability distribution defined by a probability density function, $f\left( t \right)$. The distribution function of $T$ is defined by

$F(t) = P(T &lt; t) = \int_{0}^{t} f(u)\,du \tag{1}$

and represents the probability that the survival time is less than some value $t$.

The survival function $S\left( t \right)$ indicates the probability that an individual will continue to live beyond a given time point $t$, implying that the event occurs after time $t$ [9]. It is defined by

$S(t) = P(T > t) = 1 - F(t) \tag{2}$

Hazard function

The hazard function $h\left( t \right)$ represents the likelihood that an individual will encounter an event within a specific time interval after time $t$, assuming the individual has survived up to that point. It is defined by

$h(t) = \lim_{\Delta t \to 0} \frac{P\!\left( t \le T &lt; t+\Delta t \mid T \ge t \right)}{\Delta t} \tag{3}$

The hazard function is also known as the hazard rate, intensity function, or force of mortality [8,9].

Cox proportional hazards model

The Cox proportional hazards model, developed by Cox in 1972, is the most commonly employed tool in survival analysis. It allows for estimating the effects of covariates on the hazard rate without specifying the baseline hazard function and assumes a multiplicative effect of covariates. It is given by

$h_{i}\left( t \right)= h\left( t |\textbf{x}_{i} \right) = h_{o}\left( t \right) \exp\left( \beta^{'}\textbf{x}_{\textbf{i}} \right) \tag {4}$

where $h_{i}\left( t \right)$ is the hazard rate at time $t$ for the $ith$ individual, and $h_{o}\left( t \right)$ is called the baseline hazard function, which is the hazard function for an individual whose covariates have values of 0. $\beta = \left[ \beta_{1}, \beta_{2}, ... ,\beta_{k} \right]^{'}$ is the vector of regression coefficients of the $k$ covariates and $\textbf{x}_{\textbf{i}} = \left[ x_{1i}, x_{2i}, ... , x_{ki} \right]$ is the vector of covariates or risk factors for the $ith$ individual. The model (4) can be expressed in the following form

$log\left( \frac{h\left( t|\textbf{x}_{{i}} \right)}{h_{o}\left( t \right)} \right)=\left(\beta ^{'}\textbf{x}_{i} \right) \tag{5}$

This model is based on the key assumption of proportional hazards, which suggests that the hazard ratio between two individuals with covariate values $\textbf{x}_{{i}}$ and $\textbf{x}_{{l}}$ remains unchanged over time. The model (4) can be expressed in the following form

$HR = \frac{h\left(t \mid \mathbf{x}_{i}\right)}{h\left(t \mid \mathbf{x}_{l}\right)} = \frac{h_0(t)\, \exp\left(\beta' \mathbf{x}_{i}\right)}{h_0(t)\, \exp\left(\beta' \mathbf{x}_{l}\right)} = \frac{\exp\left(\beta' \mathbf{x}_{i}\right)}{\exp\left(\beta' \mathbf{x}_{l}\right)} \tag{6}$

The model is time-independent and is therefore often referred to as the hazard ratio [10,11].

Partial likelihood

Suppose that there are no ties between event times. Let $t_{1} &lt; t_{2} &lt; \dots &lt; t_{n}$ denote ordered event times, and the Cox likelihood relies on the sequence in which events are observed rather than on their joint distribution. Thus, the Cox likelihood is called a partial likelihood and is given by

$L\left( \beta \right)=\prod_{i=1}^{n}\left[ \frac{exp\left( \beta^{'}\textbf{x}_{i} \right)}{\sum_{j\epsilon R\left( t_{i} \right)}exp\left(\beta ^{'}\textbf{x}_{j} \right)^{}} \right]^{\delta_{i}} \tag{7}$

where $R\left( t_{i} \right)$ is the risk set at time $t_{i}$ and $\delta_{i}$ is the censoring indicator such that $\delta_{i}=1$ if $t_{i}$ is an event time and $\delta_{i}=0$​​​​ if $t_{i}$ is a censored time. The corresponding log partial likelihood function is as follows

$L\left(\beta\right)=\sum_{i=1}^{n}\delta_{i}\left[\beta^{'}\textbf{x}_{i}-log\sum_{j\epsilon R\left( t_{i} \right)}exp\left( \beta^{'}\textbf{x}_{j} \right)\right] \tag{8}$

The derivative with respect to $\beta$ is given by

$\frac{\partial logl(\beta)}{\partial\beta}=\sum_{i=1}^{n}\delta_{i}\left[ \textbf{x}_{i}-\frac{\sum_{j\epsilon R\left( t_{i} \right)}^{}\textbf{x}_{jk}exp\left( \beta^{'}\textbf{x}_{j} \right)}{\sum_{j\epsilon R\left( t_{i} \right)}exp\left(\beta ^{'}\textbf{x}_{j} \right)^{}} \right]\tag{9}$

The estimator of the variance of the estimator of the coefficient is [10-12]:

$\frac{\partial logl(\beta)}{\partial\beta}=-\sum_{i=1}^{n}\left[ \frac{(\sum_{j\epsilon R\left( t_{i} \right)}exp\left( \beta^{'}\textbf{x}_{j} \right)^{})\left( \sum_{j\epsilon R\left( t_{i}\right)}^{} x_{jk}exp\left( \beta^{'}\textbf{x}_{j} \right)\right)-\left( \sum_{j\epsilon R\left( t_{i} \right)}x_{jk}exp\left( \beta^{'}\textbf{x}_{j} \right)^{2} \right)^{}}{\left( \sum_{j\epsilon R\left( t_{i} \right)}exp\left( \beta^{'}\textbf{x}_{j}\right)^{} \right)^{2}} \right]\tag{10}$

Likelihood ratio test

The likelihood ratio test is used for testing the model fitness of the Cox proportional hazards model [11,12]. The partial likelihood ratio with respect to all elements in $\left( \hat{\beta} \right)$ is defined by

$LR\left( \beta \right)=\frac{L_{p}\left( 0 \right)}{L_{p}\left( \hat{\beta} \right)}\tag{11}$

where $L_{p}\left( 0 \right)$ is the partial likelihood for the model without covariates and $L_{p}\left( \hat{\beta} \right)$ is the partial likelihood function containing all covariates. The partial likelihood ratio test statistic is given by

$LRT = 2 \log L_p(\beta) - 2 \log L_p(0) = -2 \left[ \log L_p(0) - \log L_p(\beta) \right] \tag{12}$

where

$logL_{p}\left( 0 \right)=-\sum_{i=1}^{k}log\left( n_{i} \right)$.

Schoenfeld residuals

The Schoenfeld residuals are used in evaluating the assumption of proportional hazards after fitting a Cox model. The $ith$ Schoenfeld residual for, the $jth$ explanatory variable in the fitted Cox model is given by

$r_{sji}=\delta_{i}\left[x _{ji}-\hat{a}_{ji} \right]\tag{13}$

where $\delta_{i}$ is an event indicator, which takes the value 0 if the observed survival time of the $ith$ individual is censored, and 1 if it is uncensored, $x_{ji}$ is the value of the $jth$ explanatory variable, $j=1,2, ... ,k$ for the $ith$ individual in the study,

$\hat{a}_{ji}=\frac{\sum_{l\epsilon R\left( t_{i} \right)}x_{ji}exp\left( \hat{\beta}^{'}x_{l}\right)^{}}{\sum_{l\epsilon R\left( t_{i} \right)}exp\left( \hat{\beta}^{'}x_{l}\right)^{}}\tag{14}$

where $R\left( t_{i} \right)$ is the set of all individuals at risk at time $t_{i}$ [9].

## Results

This study included 6,410 children diagnosed with malaria; males constituted 56.08% and females 43.92%. Censored cases were 95.27%, and those who died constituted 4.73%. The average age was 23.66 months (95% CI: 23.01-24.32), and the average weight was 9.93 kilograms (95% CI: 9.80-10.06 kg) (Table 1). Categorical covariates were summarized using percentages and totals.

**Table 1:** Summary statistics of continuous covariates of children diagnosed with malaria

Covariate	Mean	Standard Error	95% CI (Lower, Upper)	
Age (months)	23.66415	0.33577	23.00593	24.32237
Weight (kilograms)	9.92896	0.06609	9.79941	10.05851

The covariate “place of residence” for children diagnosed with malaria showed 4.28% of events in urban areas and 5.24% in suburban areas. The percentages of deaths were 3.78% and 5.33% in children treated with quinine and artesunate, respectively. Regarding the comorbidity status covariate, the percentages were 4.49% and 5.48% for children without and with comorbidity, respectively (Table 2). 

**Table 2:** Summary of the number of events and censored data of categorical covariates of children diagnosed with malaria

Covariate	Total	Percentage	Event	Percentage	Censored	Percentage
Gender						
Male	3595	56.08	145	4.03	3450	95.97
Female	2815	43.92	158	5.61	2657	94.39
Season						
Wet	4014	62.62	186	4.63	3828	95.37
Dry	2396	37.38	117	4.88	2279	95.12
Treatment						
Quinine	2490	38.85	94	3.78	2396	96.22
Artesunate	3920	61.15	209	5.33	3711	94.67
Place of residence						
Urban	3434	53.57	147	4.28	3287	95.72
Suburban	2976	46.53	156	5.24	2820	94.76
Comorbidity status						
With comorbidity	1552	24.21	85	5.48	1467	94.52
Without comorbidity	4858	75.79	218	4.49	4640	95.51

The covariates age, weight, treatment, and comorbidity status were statistically significant (p < 0.01), and the hazard ratio has a 95% CI that does not include 1, indicating that these covariates were significantly associated with the survival time of children with malaria. However, according to Table 3, the covariates gender, season, and place of residence were not statistically significant, as their p-values were greater than 0.05, suggesting that these covariates do not affect survival time.

**Table 3:** Cox proportional hazards model for malaria risk factors among children diagnosed with malaria Statistical test used: Wald test; significance level: p < 0.01.

Covariate	Coefficient	Std. Error	p-value	Hazard Ratio	95% CI for Hazard Ratio	
Age	0.0220963	0.0032935	0.000	1.0223420	1.0157640	1.0289630
Gender	-0.1267249	0.1151653	0.271	0.8809760	0.7029675	1.1040610
Weight	-0.1610368	0.0224883	0.000	0.8512608	0.8145552	0.8896203
Season	-0.0566143	0.1202038	0.638	0.9449585	0.7466123	1.1959980
Treatment	-0.3414782	0.1307339	0.009	0.7107189	0.5500688	0.9182877
Comorbidity status	-0.5923593	0.1315470	0.000	0.5530210	0.4273351	0.7156730
Place of residence	-0.1381185	0.1154303	0.231	0.8909955	0.6946427	1.0921200

The covariate age has a hazard ratio of 1.0223 (95% CI: 1.0158-1.0280), suggesting that for each additional month of age, the risk of death increases by 2.23%. The positive coefficient, 0.0221, of the age covariate is associated with a higher risk of death. The covariate body weight has a hazard ratio of 0.8513 (95% CI: 0.8146-0.8896), suggesting that each additional kilogram of weight reduces the risk of death by 15%. The negative coefficient, -1.1610, of the body weight covariate indicates that it is associated with increased survival time.

The covariate treatment has a hazard ratio of 0.7107 (95% CI: 0.5501-0.9183), indicating that children treated with quinine have a 29% lower hazard of death compared to those treated with artesunate. Its negative coefficient, -0.3415, shows that treatment is associated with increased survival time. Additionally, the covariate comorbidity status has a hazard ratio of 0.5530 (95% CI: 0.4273-0.7157), indicating that children without comorbidity have a 45% reduced risk of death compared to those with comorbidity. The negative coefficient, -0.5924, of the comorbidity status is associated with higher survival time.

To test the Cox proportional hazards assumption, the Schoenfeld residuals test was performed. In Table 4, the results showed that both the individual covariate and the global test were statistically insignificant (p-values > 0.05). The findings indicate that the model complies with the proportional hazards assumption, showing no signs of violation.

**Table 4:** Schoenfeld residuals test for proportional hazards assumptions Statistical test used: Chi-square test, significance level: p < 0.05.

Covariate	rho	Chi-square	df	p-value
Age	0.08394	1.25	1	0.2635
Gender	-0.04381	0.58	1	0.4468
Weight	0.00394	0.01	1	0.9395
Season	-0.02102	0.13	1	0.7170
Treatment	0.06608	1.25	1	0.2627
Comorbidity status	0.09527	2.59	1	0.1078
Place of residence	-0.01557	0.07	1	0.7865
Global test		6.72	7	0.4582

## Discussion

The findings of this study indicate that age is a significant factor in determining the survival rates of children affected by malaria. Age is positively associated with an increased risk of death among children with malaria. This result is consistent with a study by Roberts and Matthews [13] on risk factors for malaria in children in Uganda, which revealed that older children are linked to an increased risk of contracting malaria. Also, Obasohan et al. [14], in a review of studies to determine factors associated with malaria in children younger than five years in sub-Saharan Africa, found that as a child’s age increases, the odds of malaria also increase. This may be attributed to the lifestyle of children, as older ones have the freedom to move about and spend more time outside, which could make them more susceptible to mosquito bites and, as a result, malaria. Additionally, reduced survival in older children may result from delays in developing functional immunity.

However, this result contrasted with a study on severe malaria among children in the Democratic Republic of Congo, which revealed high mortality among children under five years [15], and a study by Hollowell et al. [5] on public health determinants of child malaria mortality in Kenya. Their results reveal that children of the youngest age have a higher hazard of death from malaria compared to older children. Also, Rumisha et al. [16] reported decreased survival with increasing age, implying that age is negatively related to the odds of dying, which may be because younger children are at a higher risk of death due to insufficient immunity and increased vulnerability to illnesses.

The covariate body weight has a significant influence on survival among children with malaria. The results suggest that weight is associated with increased survival in children. This result is in agreement with the studies by Amoako et al. [17] on determinants of child mortality due to malaria in Ghana, which indicated a negative relationship between weight and child death from malaria, and a study by Bekele and Kumie [18] in Ethiopia, which showed a strong association between malaria in children and weight measures. This can be attributed to the fact that improved weight status reduces the risk of malaria death and lessens the severity of malaria. Underweight children are more prone to malaria due to a compromised immune system, thus causing a high hazard of death, especially in young children.

The covariate comorbidity status showed a significant effect on child survival from malaria. Children with comorbidities have a high risk of death and decreased survival. This result is consistent with a study by Ghanghoriya et al. [19], which examined malaria and associated comorbidities among children with febrile illness in India. They found that comorbidity was significantly associated with a higher risk of death. Additionally, Mutsigiri-Murewanhema et al. [20] showed that having comorbidities significantly affects severe malaria patients and is associated with an increased hazard of death in children. Comorbidity could delay a patient’s recovery and may result in progression to severe illness and potential death. 

The covariate treatment significantly influences the survival of children with malaria. Children treated with quinine have a lower risk of death than those treated with artesunate. This result is in agreement with earlier studies by Patel et al. [21], which assessed the use of quinine versus artesunate in treating severe falciparum malaria and concluded that quinine is the preferred choice. Furthermore, Goyal et al. [22] conducted a randomized controlled trial comparing the efficacy of quinine and artesunate, finding that quinine continues to be an effective treatment for malaria.

However, this result is contrary to the finding of a study by Mayamba et al. [23], who found artesunate to be more effective than quinine in the treatment of severe malaria in children in the Democratic Republic of Congo. Furthermore, according to Sagbo et al. [24], artesunate proves to be a more effective treatment for severe malaria in children compared to quinine. This is demonstrated by a reduction in both the duration of treatment and the mortality risk in Benin.

The contradiction in the treatment outcome may be attributable to several factors, including differences in practices, criteria applied for severe malaria, noncompliance with treatment, or inadequate or inappropriate administration of artesunate. In addition, parasite drug resistance, poor-quality drugs, frequent malaria transmission, and the disease remain consistently prevalent.

This study has certain limitations: being a single-center cohort study, its findings might not accurately represent the wider population. The design of a retrospective study relies on hospital records, which might have incomplete data, potentially leading to biases that could affect the results.

## Conclusions

This study found that age and comorbidity status are associated with an increased risk of death among children with malaria. In contrast, increased body weight reduces the hazard of death. Also, treatment with quinine is associated with a reduced risk of death compared to artesunate, which is contrary to studies showing artesunate to be superior to quinine. Quinine appears to be more effective in the management of malaria in South Sudan. Enhancing programs aimed at reducing the malaria burden in children and ensuring the availability of good-quality drugs to improve treatment outcomes is important for child health. It is also recommended to conduct a study comparing quinine and artesunate for the treatment of malaria. Multi-center and prospective cohort studies should be considered in future research on malaria in South Sudan to address issues of generalizability and incomplete data.
